# Low-Level Laser Therapy in the Management of Bisphosphonate-Related Osteonecrosis of the Jaw

**DOI:** 10.3390/jcm14134441

**Published:** 2025-06-23

**Authors:** Vincenzo Bitonti, Rocco Franco, Lorenzo Cigni, Domenico Familiari, Gioele Gravili, Giuseppe Vazzana, Pietro di Michele

**Affiliations:** UOC Odontostomatologia e Chirurgia Orale, AUSL Modena, 41122 Modena, Italy; v.bitonti@ausl.mo.it (V.B.); l.cigni@ausl.mo.it (L.C.); d.familiari@ausl.mo.it (D.F.); g.gravili@ausl.mo.it (G.G.); g.vazzana@ausl.mo.it (G.V.); p.dimichele@ausl.mo.it (P.d.M.)

**Keywords:** bisphosphonate-related osteonecrosis of the jaw (BRONJ), low-level laser therapy (LLLT), systematic review

## Abstract

**Background**: Bisphosphonate-Related Osteonecrosis of the Jaw (BRONJ) is a severe complication associated with bisphosphonate therapy, commonly used in the treatment of osteoporosis and metastatic bone diseases. Low-Level Laser Therapy (LLLT) has been proposed as a potential treatment modality for BRONJ, with its anti-inflammatory, analgesic, and regenerative effects being of particular interest. This systematic review aims to critically assess the current evidence regarding the efficacy of LLLT in the management of BRONJ. **Methods**: This review was conducted following the PRISMA (Preferred Reporting Items for Systematic Reviews and Meta-Analyses) guidelines. A comprehensive search of electronic databases, including PubMed, Scopus, and Web of Science, was performed to identify relevant studies published up to September 2024. The systematic review protocol has been registered on the International Prospective Register of Systematic Reviews (PROSPERO) with the number 423003. All studies considered are observational. Studies were included if they investigated the application of LLLT in BRONJ treatment, reporting clinical outcomes such as pain reduction, lesion healing, and quality of life. The quality of the studies was assessed using the Cochrane Risk of Bias tool, and the data were synthesized descriptively. **Results**: A total of four studies met the inclusion criteria. The findings indicate that LLLT, particularly when used in conjunction with surgical debridement and pharmacological therapy, significantly may reduce pain and promote soft tissue healing in patients with BRONJ. However, the heterogeneity of study designs, laser parameters, and outcome measures limits the generalizability of these results. Furthermore, most studies were small-scale, with moderate to high risk of bias. **Conclusions**: The current evidence suggests that LLLT may be a beneficial adjunctive therapy in the treatment of BRONJ. However, conclusions are limited by the lack of randomized controlled trials and methodological heterogeneity, particularly for pain management and soft tissue regeneration. However, further high-quality randomized controlled trials with standardized laser protocols are necessary to establish its efficacy and optimize clinical application. Therefore, one of the limitations of this literature review with meta-analysis is that only four studies were considered and, moreover, they were observational. The results of the meta-analysis showed that there is not enough evidence to declare a statistical correlation; this result will surely be due to the small number of studies and heterogeneity.

## 1. Introduction

Bisphosphonate-related osteonecrosis of the jaw (BRONJ) is a debilitating condition characterized by exposed necrotic bone in the maxillofacial region, typically associated with the administration of bisphosphonates, a class of drugs widely used to treat bone-related diseases such as osteoporosis, Paget’s disease, and bone metastases from cancer.

Bisphosphonate-related osteonecrosis of the jaw (BRONJ) is a serious and relatively uncommon complication associated with the use of bisphosphonate medications. It is defined clinically as the presence of exposed necrotic bone in the maxillofacial region that persists for more than eight weeks in a patient who is currently receiving or has previously received bisphosphonate therapy, and who has no history of radiation therapy to the craniofacial region or evidence of metastatic disease in the jaw.

While bisphosphonates effectively inhibit osteoclastic bone resorption, their long-term use has been linked to complications in bone healing, leading to the development of osteonecrosis in the jaw, a condition that significantly impairs the quality of life. The exact pathophysiology of BRONJ remains under investigation, but several mechanisms, such as the inhibition of osteoclastic activity, impairment of angiogenesis, and microbial infections, have been proposed as contributing factors. BRONJ is a challenging clinical condition to manage due to its refractory nature, and despite numerous treatment protocols, an optimal therapeutic approach has not been established [1,2]. There are no studies that consider non-conventional techniques for the treatment of osteonecrosis, other than the surgical and pharmacological approach. The etiology of BRONJ is multifactorial and involves an interplay of pharmacologic, local, and systemic factors. Bisphosphonates, particularly the nitrogen-containing variants, exert their therapeutic effects by inhibiting osteoclast-mediated bone resorption, which leads to reduced bone turnover. While beneficial in conditions characterized by excessive bone loss, this suppression of remodeling can impair the bone’s ability to recover from injury or infection. Patients undergoing invasive dental procedures, such as extractions or implant placements, are at particular risk, as these interventions can disrupt the alveolar bone and initiate a cascade of events that may lead to necrosis in the context of compromised bone metabolism. Additional risk factors include local infection, poor oral hygiene, systemic conditions such as diabetes, and the concurrent use of corticosteroids or chemotherapeutic agents, all of which may contribute to a higher susceptibility to BRONJ.

The pathogenesis of BRONJ is not entirely understood, but several mechanisms have been proposed. A primary factor is the inhibition of normal bone remodeling, which prevents the repair of microdamage and leads to bone fragility over time. This is compounded by the antiangiogenic properties of bisphosphonates, which may impair the vascular supply to the jawbones, reducing tissue perfusion and limiting the capacity for healing. Furthermore, the oral cavity harbors a complex microbial environment, and the presence of exposed bone can allow for bacterial colonization, leading to persistent inflammation and secondary infection. Soft tissue toxicity has also been implicated, as bisphosphonates may negatively affect the viability of oral mucosal cells, thereby impairing wound healing and the re-establishment of an effective mucosal barrier. In addition, mechanical trauma from routine oral function or ill-fitting prostheses can contribute to the development or exacerbation of lesions in at-risk individuals.

The clinical presentation of BRONJ varies, ranging from asymptomatic bone exposure to severe pain, infection, and even pathological fractures in advanced cases. Currently, management strategies include conservative approaches, such as antibiotic therapy, oral rinses, and analgesics, as well as more invasive interventions like surgical debridement and resection of necrotic bone [3]. However, the outcomes of these treatments are often unpredictable, and recurrence rates remain high. The chronic nature of BRONJ and the impact of treatment on patients’ well-being necessitate the exploration of adjunctive therapies that can enhance healing, alleviate symptoms, and improve overall clinical outcomes [4,5,6]. Treatment approaches depend on the severity of the condition and underlying risk factors. In the early stages, conservative management with antibiotics, antiseptic mouth rinses, and pain control can help prevent progression. For moderate cases, surgical debridement may be necessary to remove necrotic bone and reduce complications. In severe instances, more extensive procedures like bone resection and reconstruction with grafts or implants might be required to restore function. Additionally, adjunctive therapies such as hyperbaric oxygen therapy, platelet-rich plasma, and low-level laser therapy are being explored to enhance healing and bone regeneration.

There is growing scientific interest in the potential role of low-level laser therapy (LLLT)—also known as photobiomodulation therapy (PBMT)—as a supportive treatment in the prevention and management of bisphosphonate-related osteonecrosis of the jaw (BRONJ).

The connection between BRONJ and LLLT lies in the biological effects of laser therapy on wound healing, inflammation modulation, angiogenesis, and bone regeneration, all of which are impaired in the pathogenesis of BRONJ. Since bisphosphonates suppress bone remodeling and angiogenesis—two essential processes for bone and mucosal healing—LLLT may help counteract these effects through its ability to stimulate cellular activity and improve tissue repair.

Several studies have demonstrated that LLLT can enhance osteoblast function, increase vascular endothelial growth factor (VEGF) expression, and promote fibroblast proliferation and collagen synthesis. These effects are particularly relevant in the jaw, where the compromised healing environment due to bisphosphonates leads to prolonged exposure of necrotic bone. By improving local microcirculation, reducing oxidative stress, and enhancing epithelial closure, LLLT may support faster mucosal recovery and reduce the risk or severity of bone exposure.

Clinically, LLLT has been investigated both as a preventive adjunct—applied before and after dental surgery in patients on bisphosphonate therapy—and as a therapeutic intervention in established BRONJ cases.

In recent years, low-level laser therapy (LLLT) has gained attention as a potential non-invasive treatment modality for a variety of conditions, including musculoskeletal disorders, wound healing, and dental diseases. LLLT, also known as photobiomodulation, involves the application of low-intensity laser light to tissues to induce biological effects at the cellular level. The proposed mechanisms of action for LLLT include the stimulation of mitochondrial activity, leading to increased adenosine triphosphate production, modulation of reactive oxygen species, and activation of transcription factors involved in cellular proliferation and repair. These effects are thought to contribute to the anti-inflammatory, analgesic, and regenerative properties of LLLT, making it a promising option for treating conditions characterized by impaired tissue healing, such as BRONJ [7,8,9,10,11,12,13,14,15,16,17,18].

Several studies have investigated the application of LLLT in the management of BRONJ, with a focus on its ability to reduce pain, promote soft tissue healing, and enhance bone regeneration [19,20]. Initial findings suggest that LLLT may have a beneficial role as an adjunctive therapy, particularly when used in combination with conventional treatments like surgical debridement and pharmacological interventions. However, despite the growing interest in this therapeutic approach, the lack of standardized laser parameters complicates the comparability of findings [21,22,23]. The variation in study designs, laser parameters, treatment protocols, and outcome measures complicates the interpretation of results, and the lack of large-scale, high-quality randomized controlled trials (RCTs) further limits the generalizability of current findings.

The potential of LLLT to modulate the inflammatory response and promote tissue regeneration is particularly relevant to the treatment of BRONJ, where chronic inflammation and impaired wound healing are central features of the disease process. By reducing the inflammatory burden and enhancing cellular repair mechanisms, LLLT could theoretically mitigate the progression of BRONJ and improve clinical outcomes. Additionally, the analgesic effects of LLLT offer a valuable benefit in managing the often significant pain experienced by patients with BRONJ, which can otherwise be difficult to control with conventional analgesic medications. This dual action of LLLT—targeting both pain and tissue healing—positions it as a potential comprehensive treatment modality for BRONJ, addressing two of the most challenging aspects of the condition [24,25,26,27].

This systematic review, conducted in accordance with the PRISMA (Preferred Reporting Items for Systematic Reviews and Meta-Analyses) guidelines, aims to critically evaluate the current evidence on the effectiveness of LLLT in the management of BRONJ. By synthesizing data from clinical studies, we seek to determine the extent to which LLLT can improve clinical outcomes for patients with BRONJ and identify any gaps in the existing research that need to be addressed in future studies. Specifically, this review will explore the impact of LLLT on key clinical outcomes, such as pain reduction, soft tissue healing, bone regeneration, and overall quality of life in patients with BRONJ.

In addition to assessing the efficacy of LLLT, this review will also examine the safety profile of this treatment, as well as any potential contraindications or adverse effects reported in the literature. The goal is to provide clinicians with a comprehensive overview of the current state of knowledge regarding LLLT in BRONJ and to offer evidence-based recommendations for its use in clinical practice [28,29,30,31,32,33,34,35,36,37,38,39].

Laser treatment was initially introduced as a therapeutic strategy to encourage tissue regeneration of damaged tissues by Mester et al. [40]. A novel approach, low-level laser treatment (LLLT), has been demonstrated to have a number of beneficial outcomes, such as neuron regeneration, wound healing, and pain alleviation. Furthermore, it may have antibacterial and biostimulating properties when applied to oral tissue, reducing edema following the extraction of the third molar [41], decreasing oral mucositis [42], and enhancing wound healing [43,44] and epithelization following periodontal surgery [45,46]. LLLT may be useful in treating medication-related osteonecrosis of the jaw because of these factors [47,48,49,50,51].

The findings from this systematic review will contribute to a better understanding of the potential benefits and limitations of LLLT in the treatment of BRONJ and help guide future research efforts aimed at optimizing the management of this challenging condition. This systematic review aims to critically assess the current evidence regarding the efficacy of LLLT in the management of BRONJ.

## 2. Materials and Methods

### 2.1. Eligibility Criteria

All documents were assessed for eligibility based on the Population, Exposure, Comparator, and Outcomes (PECO) model [52].

(P) Participants consisted of human subjects.

(E) The Exposure consisted of patients with BRONJ treated with LLLT.

(C) The Comparison was with the control group treated with other therapy.

(O) The Outcome consisted of evaluating the efficacy and the incidence of complications between patients treated with surgery and/or antibiotic therapy and others also treated with LLLT.

Only papers providing data at the end of the intervention were included. Exclusion criteria were as follows: (1) patients with head and neck radiotherapy; (2) full-text unavailability (i.e., posters and conference abstracts); (3) studies involving animals; (4) review (topical or systematic) articles; (5) case reports/series.

### 2.2. Search Strategy

We systematically searched the Web of Science, PubMed, and Scopus for articles published from the inception until September 2024, following the strategy reported in Table 1. Furthermore, we manually searched the references.

This systematic review was conducted according to the guidance of the Cochrane Handbook for Systematic Reviews of Interventions and the Preferred Reporting Items for Systematic Reviews (PRISMA) guidelines 2020 (Supplementary Materials). Gray literature was not considered in this review. The systematic review protocol has been registered on the International Prospective Register of Systematic Reviews (PROSPERO) with the number 423003.

### 2.3. Data Extraction

Two reviewers (RF, VB) extracted the data from the included studies using customized data extraction on a Microsoft Excel sheet. In cases of disagreement, a consensus was reached through a third reviewer (GV). The third reviewer read the article in full and decided whether to include it among the eligibility criteria. The following data were extracted: (1) title; (2) research question; (3) main findings; (4) authors; (5) number of patients; (6) BRONJ sites; (7) treatment modalities; (8) key findings.

### 2.4. Quality Assessment

For this review, the Risk of Bias in Non-randomized Studies of Interventions (ROBINS-E) tool was utilized to evaluate potential biases in the included studies. This tool provides a detailed framework to systematically assess bias in non-randomized research. Each study underwent independent scrutiny by at least two trained reviewers who adhered to the ROBINS-E guidelines. The evaluation covered seven critical domains: confounding factors, participant selection, intervention classification, deviations from intended treatments, missing data, outcome measurement, and selective reporting of results. To ensure unbiased and consistent assessments, any disagreements between reviewers were addressed through discussion and consensus. If no agreement was reached, a third reviewer intervened to make the final decision. This process facilitated a comprehensive examination of potential biases in the studies, highlighting both their strengths and weaknesses. Assessing the risk of bias allowed the authors to better understand the reliability of the evidence and draw conclusions based on a more informed interpretation of the findings.

### 2.5. Statistical Analysis

The data analysis was carried out using the Review Manager software (RevMan), version 5.2.8, developed by the Cochrane Collaboration (Copenhagen, Denmark; 2014). The study focused on determining the effects of low-level laser therapy (LLLT) in managing bisphosphonate-related osteonecrosis of the jaw (BRONJ). Mean differences between intervention and control groups were calculated. Study heterogeneity was assessed using the Higgins *I*^2^ statistic and the chi-square test, with thresholds defined as low (<30%), moderate (30–60%), or high (>60%) heterogeneity.

### 2.6. Evidence Quality Assessment

To evaluate the reliability of the evidence and the level of certainty regarding the findings, the Grading of Recommendations Assessment, Development, and Evaluation (GRADE) approach was employed. This framework helped classify the strength of evidence and provided insight into the confidence level associated with the results of the review [53].

## 3. Results

### 3.1. Study Characteristics

**Figure 1:**
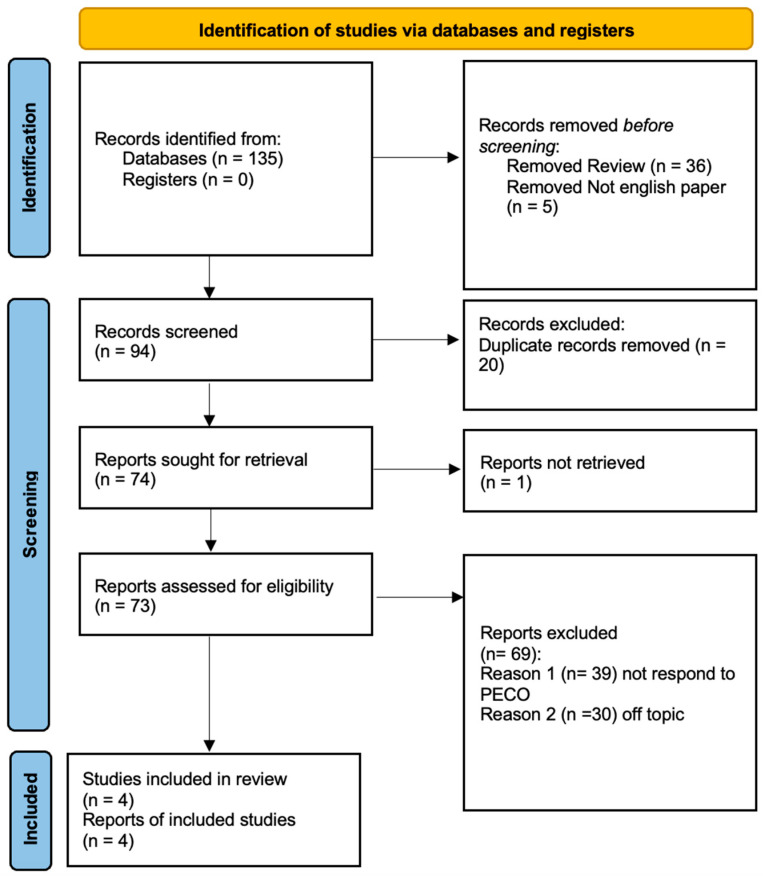
Prisma flowchart of the included studies.

A total of 135 studies were identified through searches conducted across three databases. During the initial phase, 20 duplicate records were removed, and 5 studies were excluded, as they were not written in English. In the first screening step, 36 articles were discarded because they were systematic reviews and did not align with the inclusion criteria. Additionally, only randomized clinical trials were considered by applying a specific filter. In the final stage, 73 articles underwent evaluation, including both abstract and full-text reviews. Out of these, only 4 studies were selected for the systematic review, as depicted in the PRISMA 2020 flowchart (Figure 1). The remaining 69 studies were excluded, with 39 failing to meet the PECO criteria and 30 being irrelevant to the topic. Titles and abstracts were screened according to the PECO framework, resulting in 4 articles being deemed eligible and included in the final review from the databases searched.

### 3.2. Main Findings

The study of Vescovi et al. (2010) [54] involved a cohort of patients who underwent various interventions, including surgical debridement and laser-assisted techniques, specifically utilizing Er:YAG lasers. The findings indicate that laser-assisted treatments significantly improved healing outcomes compared to conventional surgical methods. Specifically, the study reported a complete mucosal healing rate of 87.5% among patients treated with laser techniques, with many patients transitioning from higher stages of BRONJ to lower stages post-treatment. Statistical analyses demonstrated that the use of Er:YAG lasers, in conjunction with LLLT, resulted in a significant improvement in the healing of necrotic bone sites (*p* < 0.0001) [54]. The second study was by Vescovi et al. (2012) [55]. The patients were grouped into four categories based on the treatment they received: Group 1 (G1) included those treated with antibiotics only; Group 2 (G2) involved patients treated with antibiotics and LLLT; Group 3 (G3) received antibiotics and surgical treatment; and Group 4 (G4) received antibiotics, surgical treatment, and LLLT. Treatments were performed at the Unit of Oral Pathology and Medicine and Laser-Assisted Surgery of the University of Parma between January 2004 and July 2009. BRONJ sites were classified according to the American Association of Oral and Maxillofacial Surgeons’ staging system. Various factors, including additional risk factors such as smoking, diabetes, and corticosteroid use, were uniformly distributed across the groups. Antibiotic treatment involved amoxicillin and metronidazole, while LLLT was performed using a Nd:YAG laser. Surgical interventions varied based on lesion severity, involving mucoperiosteal flap elevation and bone removal via conventional burs or an Er:YAG laser. The efficacy of treatments was assessed by clinical improvement, defined as the transition to a lower BRONJ stage for at least six months. The results of the study showed that clinical improvement varied across the different treatment groups. In Group 1 (G1), which received only antibiotic therapy, 25% of the BRONJ sites (3 out of 12) showed improvement, with 16.6% reaching complete mucosal healing (stage 0). Group 2 (G2), which received antibiotics combined with LLLT, had 66.6% of the sites (18 out of 27) showing improvement, with 33.3% achieving stage 0. Group 3 (G3), which involved antibiotics and surgical therapy, showed improvement in 52.9% of the sites (9 out of 17), all of which reached stage 0. The highest improvement rate was observed in Group 4 (G4), which combined antibiotics, surgical therapy, and LLLT. In this group, 88.8% of the sites (40 out of 45) improved, and 73.3% achieved complete healing (stage 0). Statistically significant differences were found between the outcomes of G1 and G2 (*p* = 0.0346), as well as between G4 and the other groups (*p* < 0.05). A comparison of medical (G1 + G2) versus surgical (G3 + G4) approaches showed that surgical treatments had better outcomes in terms of complete healing and clinical improvement. Additionally, laser-assisted treatments (G2 + G4) had higher clinical improvement rates than non-laser-assisted treatments (G1 + G3) (*p* = 0.0003). The location of the lesions (mandible or maxilla) and the underlying pathology (multiple myeloma, bone metastasis, or osteoporosis) did not significantly affect treatment outcomes [55]. The study of Vescovi et al. (2012) [56] evaluated 151 patients (110 women and 41 men) with a mean age of 67.3 and 66 years, respectively, who were diagnosed with bisphosphonate-related osteonecrosis of the jaw (BRONJ). The patients were treated at the University of Parma, Italy, from January 2004 to July 2010. Out of 192 affected bone sites, 139 were treated using different methods. The study population included patients treated with bisphosphonates (BPs) for multiple myeloma, bone metastasis, and osteoporosis. Treatment modalities were classified into five groups based on different combinations of medical therapy, LLLT, conventional surgical therapy, and laser-assisted surgery using an Er:YAG laser. The medical therapy regimen included antibiotics and antiseptic mouth rinses. LLLT was applied weekly for two months, while surgical procedures ranged from conservative debridement to laser vaporization of necrotic bone. Treatment outcomes were assessed through clinical examinations and statistical analysis of factors like BRONJ stage and primary disease. The results revealed significant differences in clinical improvement and healing across treatment groups. Clinical improvement was highest in the group treated with Er:YAG laser surgery and LLLT (96.55%), followed by medical and conventional surgical therapy combined with LLLT (81.81%), and medical therapy alone showed the lowest improvement (25%). Complete healing rates followed a similar trend, with the highest healing observed in the Er:YAG laser group (89.65%). Statistical analysis showed no correlation between primary disease and treatment response, but a significant correlation was found between BRONJ stage and treatment responsiveness. Early-stage BRONJ responded better to treatments, and surgical approaches, particularly laser-assisted, yielded the best outcomes in terms of clinical improvement and healing [56].

The study of Atalay et al. (2011) [57] evaluated 20 patients treated for bisphosphonate-related osteonecrosis of the jaw (BRONJ) at Istanbul University. The patients, 7 males and 13 females, had BRONJ affecting either the mandible or maxilla and were treated with intravenous zoledronic acid (Zometa) for cancer. Patients were divided into two groups: 10 treated with laser surgery and 10 with conventional surgery. Prior to surgery, serum C-telopeptide (CTX) levels were measured to assess bone turnover. Patients with low CTX values were given a drug holiday before surgery. Treatment outcomes, wound healing, and osteonecrosis were monitored for six months. Surgical techniques included removal of necrotic tissue and closure with sutures, and patients received postoperative antibiotics and chlorhexidine rinses.

**Figure 2:**
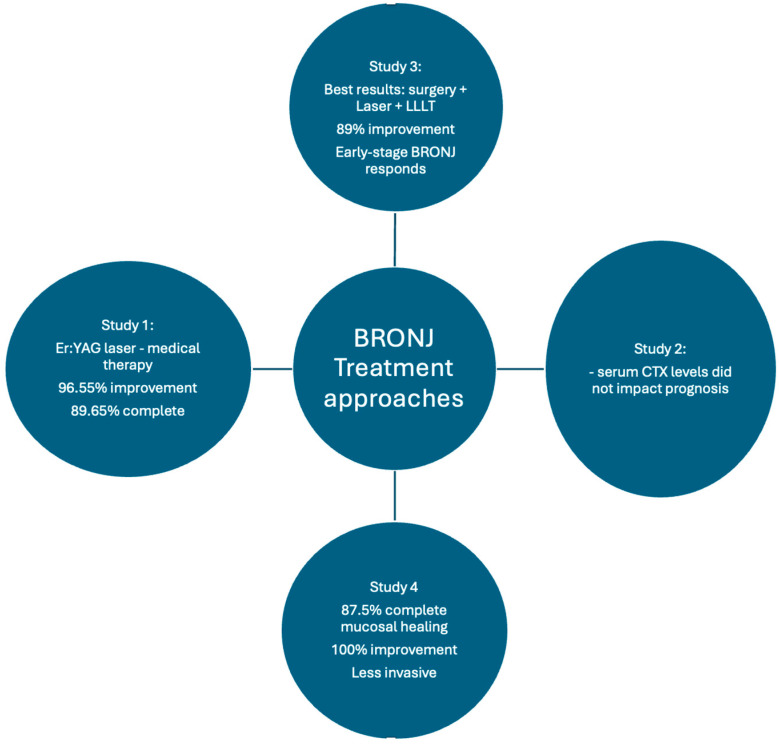
Graphical summary of the studies included in the meta-analysis. This graph concisely and visually summarizes the results of the studies. Legend: Study 1: Bisphosphonate-Related Osteonecrosis of the Jaw (BRONJ) Treatments; Study 2: Bisphosphonate-related osteonecrosis: laser-assisted surgical treatment or conventional surgery?; Study 3: Early Surgical Laser-Assisted Management of Bisphosphonate-Related Osteonecrosis of the Jaws (BRONJ): A Retrospective Analysis of 101 Treated Sites with Long-Term Follow-Up; Study 4: Surgical approach with Er: YAG laser on osteonecrosis of the jaws (ONJ) in patients under bisphosphonate therapy (BPT).

The results showed no significant differences between the laser and conventional surgery groups in terms of healing outcomes. Complete healing was achieved in 70% of patients in the laser group and 40% in the conventional group, but the difference was not statistically significant. Similarly, CTX levels did not significantly affect healing outcomes. Patients with stage II BRONJ had significantly better healing outcomes compared to those with stage I. Overall, laser surgery was shown to be a beneficial alternative; however, more research is required to further evaluate treatment protocols [57] (Table 2 and Table 3, Figure 2).

### 3.3. Meta-Analysis

**Figure 3:**
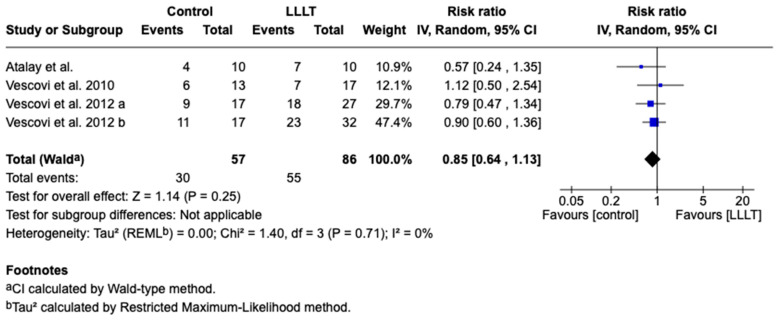
Forest plot of the included studies using random-effect model; LLLT (low-level laser therapy) [54,55,56,57]. The results show no significant difference. Note: Result not statistically significant (*p* = 0.25); interpretation should be cautious.

The meta-analysis was conducted by a random-effect model because of the low heterogeneity (*I*^2^ = 0%) among the four included studies that compared the treatment of BRONJ with LLLT and with surgery. The overall effect, reported in the forest plot (Figure 3), revealed that there is insufficient evidence to declare a statistically significant difference between LLLT and comparator treatments (RR 0.84 95% CI: 0.63–1.13; Z = 1.16; *p* = 0.25).

### 3.4. Quality Assessment and Risk of Bias of Included Articles

**Figure 4:**
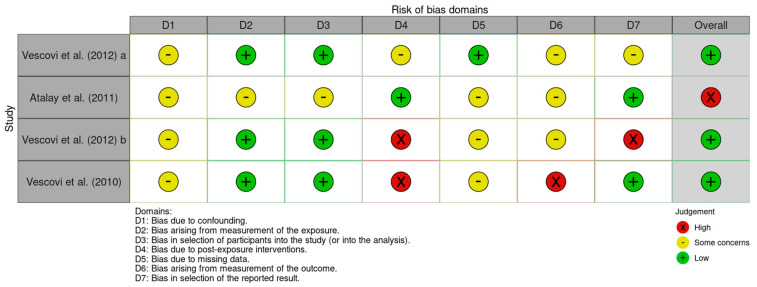
Bias assessment evaluated by ROBINS-E [54,55,56,57]. The most important section to evaluate is D6, reflecting the fact that different measurements were made regarding the control groups, and therefore, this result is reflected in this way.

Figure 4 presents the risk of bias identified in the included studies. Many studies showed some concerns regarding confounding bias. In contrast, bias related to measurement was found to be low. Participant selection also demonstrated a low risk of bias in a majority of studies. Due to significant heterogeneity among the studies, it was not possible to calculate bias associated with post-exposure factors. The risk of bias from missing data was minimal in most cases, as was the bias arising from the measurement of outcomes. However, a high level of bias was observed in the selection of reported results for the majority of studies. Ultimately, the findings indicate that four studies had a low overall risk of bias. The quality of the evidence, as assessed in Table 4, suggests moderate confidence in the findings; however, methodological limitations persist.

## 4. Discussion

BRONJ remains a significant complication in patients undergoing bisphosphonate therapy, especially for conditions like osteoporosis or metastatic bone disease. Conventional treatments for BRONJ often involve conservative management, surgical intervention, and adjunctive pharmacological approaches, but their effectiveness varies, particularly in advanced stages. In this context, LLLT offers an innovative, minimally invasive therapeutic option that leverages its anti-inflammatory, biostimulatory, and analgesic properties [58]. The implementation of LLLT in clinical practice requires a thorough understanding of the optimal laser parameters, including wavelength, energy density, and treatment duration, to maximize its therapeutic effects while minimizing potential risks. The heterogeneity observed in the current literature regarding these parameters highlights the need for standardized treatment protocols that can be universally applied across different clinical settings. Furthermore, it is essential to assess the long-term effects of LLLT on bone health, particularly in patients undergoing bisphosphonate therapy, to ensure that the benefits of this treatment outweigh any potential adverse outcomes [59]. Given the growing burden of BRONJ in patients receiving bisphosphonate therapy and the limited success of conventional treatments, it is imperative to explore innovative approaches like LLLT that may offer new hope for patients affected by this debilitating condition [60,61,62,63,64,65]. The results of this study’s pooled meta-analysis did not confirm a statistically significant benefit of LLLT as an adjunct to standard treatment protocols, even though some included studies reported improved healing outcomes. These findings align with previous research highlighting LLLT’s capacity to enhance wound healing, reduce pain, and modulate inflammatory responses at the cellular level by promoting mitochondrial activity and tissue repair. The biostimulatory effect of LLLT is particularly critical in the management of BRONJ, where compromised bone healing is a major challenge due to the inhibitory effects of bisphosphonates on osteoclast function and bone remodeling [66,67,68].

However, the interpretation of these results should consider several factors. First, while the study showed statistically significant improvements in clinical outcomes such as pain reduction and lesion resolution, the long-term effects of LLLT on bone regeneration in BRONJ remain underexplored. Future studies should prioritize long-term outcomes, consistent LLLT protocols, and blinding to reduce performance bias. More extensive clinical trials with larger patient populations and extended follow-up periods are needed to substantiate these findings and determine the sustained efficacy of LLLT over time [69].

Another consideration is the variability in treatment protocols for LLLT, including differences in wavelength, dosage, and duration of treatment across studies. Standardizing these parameters is essential for comparing results and ensuring reproducibility. Additionally, patient-specific factors, such as the stage of BRONJ, underlying systemic conditions, and individual responses to bisphosphonates, may influence the outcomes of LLLT and should be accounted for in future research [70].

While the study contributes valuable evidence supporting the role of LLLT as a complementary treatment for BRONJ, it also underscores the need for multidisciplinary collaboration in managing this condition. The integration of LLLT with other therapeutic modalities, such as pharmacological agents, surgical debridement, and systemic health management, may enhance overall treatment outcomes [71,72].

The management of bisphosphonate-related osteonecrosis of the jaw (BRONJ) represents a significant clinical challenge, particularly given the complexities associated with its etiology and treatment options [73,74,75,76]. The papers reviewed provide a comprehensive overview of the surgical approaches, particularly the use of Er:YAG laser technology and the implications of bisphosphonate therapy on jaw health. The use of Er:YAG laser in the surgical management of BRONJ has shown promising results. Specifically, studies indicate that combining laser surgery with LLLT significantly enhances mucosal healing outcomes. One study reported a complete mucosal healing rate of 87.5% in patients treated with this combined approach, underscoring the efficacy of laser-assisted techniques in promoting recovery from BRONJ [54]. This is particularly relevant given that traditional surgical methods often result in slower healing and higher rates of postoperative complications [56]. The laser’s ability to promote revascularization and reduce inflammation is critical, as these factors are essential for effective healing in osteonecrotic tissues [56]. Moreover, the literature suggests that the timing of surgical intervention plays a crucial role in treatment success. Late surgical management of BRONJ can lead to disease progression, highlighting the importance of early diagnosis and intervention [55]. The findings emphasize that conservative treatments, such as antibiotic therapy, are often insufficient in advanced stages of BRONJ, necessitating surgical options for effective management. This aligns with the broader understanding that BRONJ is not solely a consequence of bisphosphonate therapy but is influenced by various factors, including the type of bisphosphonate, duration of therapy, and patient comorbidities. The discussion of the etiology of BRONJ also warrants attention. The pathogenesis of BRONJ is multifactorial, with bisphosphonates inhibiting bone turnover and impairing the healing of microdamage, which can predispose patients to osteonecrosis. Furthermore, the risk of developing BRONJ is significantly heightened in patients undergoing invasive dental procedures while on bisphosphonate therapy, with an estimated sevenfold increase in risk for those receiving intravenous formulations. This necessitates a careful screening process and preventive dental care prior to initiating bisphosphonate therapy, particularly in patients with a history of dental issues or those requiring surgical interventions. In conclusion, the integration of Er:YAG laser technology in the surgical management of BRONJ presents a promising avenue for improving patient outcomes. The evidence supports the notion that early intervention, coupled with advanced surgical techniques, can lead to significant improvements in healing and quality of life for affected patients. Future research should focus on establishing standardized treatment protocols and further elucidating the underlying mechanisms of BRONJ to optimize management strategies [77].

A total of 128 patients diagnosed with bisphosphonate-related osteonecrosis of the jaw (BRONJ) were evaluated in the study of Vescovi, comprising 33 males and 95 females. The patient cohort included 52 individuals with multiple myeloma, 53 with bone metastases, and 23 with osteoporosis. The majority of necrotic bone cases were localized in the mandible (approximately 65%), with maxillary involvement reported in about 26% of cases, and 9% exhibiting both mandibular and maxillary involvement. The treatment modalities varied, with a focus on comparing surgical and nonsurgical approaches, particularly the use of Er:YAG laser-assisted surgery. The results indicated that patients undergoing laser-assisted surgical treatment demonstrated significantly improved healing outcomes. Specifically, complete mucosal healing was achieved in 87.5% of patients treated with the Er:YAG laser combined with LLLT, compared to lower healing rates in those receiving conventional surgical interventions [55]. This finding underscores the effectiveness of laser technology in promoting tissue regeneration and reducing recovery time. Histopathological evaluations revealed the presence of non-vital bone fragments accompanied by bacterial colonies and inflammatory cells, suggesting a complex interplay between infection and necrosis in BRONJ pathophysiology. The study also noted that patients in earlier stages of BRONJ (stages I and II) were more likely to achieve healing through antimicrobial treatment alone, while those in advanced stages required surgical intervention [57]. Furthermore, the study highlighted the challenges associated with surgical debridement, particularly the difficulty in visualizing necrotic bone borders due to limitations in imaging techniques. Despite these challenges, the use of Er:YAG laser technology facilitated more precise surgical debridement, allowing for effective removal of necrotic tissue while preserving healthy surrounding structures. In terms of overall treatment outcomes, the combination of surgical debridement and laser therapy not only improved mucosal healing rates but also minimized the need for more extensive resections, thereby preserving the quality of life for patients [57]. The results suggest that early intervention with laser-assisted techniques can significantly alter the prognosis for patients suffering from BRONJ, emphasizing the need for timely diagnosis and treatment initiation [55]. In conclusion, the findings from this study support the efficacy of Er:YAG laser-assisted surgery as a promising treatment modality for BRONJ, particularly in the earlier stages of the disease. The integration of advanced laser technologies into clinical practice may enhance healing outcomes and improve the overall management of this complex condition.

### Limitations

This systematic review and meta-analysis present certain limitations that should be acknowledged. Firstly, the number of included studies was relatively small, limiting the generalizability of the findings. Moreover, the studies are not randomized, but observational, thus making it difficult to conceptualize and make clinical the results of the meta-analysis. Additionally, the studies exhibited considerable heterogeneity in terms of patient selection criteria, laser parameters, and treatment protocols, which may have influenced the reported outcomes. An important limitation of this meta-analysis is that there are no studies that have equal comparators, so we used control groups with different therapies. However, also given the small number of studies, we could not perform a subgroup analysis to limit and exclude confounding factors. Therefore, the few studies render the possibility of the presence of bias or confounding factors that may have skewed the results of the meta-analysis. The risk of bias was moderate to high in most studies, further impacting the reliability of the results. Another limitation is the short follow-up period in many studies, which prevents a comprehensive assessment of the long-term effects of LLLT in BRONJ management. Finally, the lack of large-scale randomized controlled trials highlights the need for further research to establish standardized treatment protocols and optimize the clinical application of LLLT.

This study with a meta-analysis of the literature analyzes the effects of LLLT on the treatment of osteonecrosis. However, this review presents some problems regarding the scarcity of the present literature. We were able to analyze only four studies, found on the main search engines. Furthermore, the studies present in the literature are not RCTs that allow a comparison and a statistical analysis, free from bias. Furthermore, another problem highlighted is the lack of a homogeneous control group, which would allow for a comparison with the LLLT with a single technique. All this has created bias in the statistics. Furthermore, it was not possible to perform a subgroup analysis in order to eliminate possible confounding factors. Finally, however, we noted an improvement, not statistically significant, in the use of LLLT. Although a statistical analysis with a random-effect model was performed, the heterogeneity results were 0. This fact will surely be explained by the small number of studies and the small sample size, which make the statistics possibly biased. Therefore, further clinical studies will be necessary to be able to perform a detailed and accurate meta-analysis.

## 5. Conclusions

The results of this study provide a strong foundation for considering LLLT as an adjunctive therapy in BRONJ management. Nonetheless, further research is essential to optimize treatment protocols, assess long-term outcomes, and explore the potential of LLLT in combination with other therapeutic approaches for comprehensive BRONJ care.

In conclusion, this systematic review and meta-analysis suggest that low-level laser therapy (LLLT) may serve as a potentially beneficial adjunctive treatment; however, current evidence is insufficient to support definitive clinical recommendations. The findings support the potential of LLLT in improving clinical outcomes when used in combination with conventional treatments such as surgical debridement and pharmacological therapy. However, the overall quality of evidence remains limited due to the small number of studies, heterogeneity in treatment parameters, moderate to high risk of bias in most investigations, and the fact that the included studies are all observational. The results of this meta-analysis are greatly affected by the heterogeneity of the control groups, as they do not use the same techniques. This caused the meta-analysis to have possible confounding factors. Unfortunately, a subgroup analysis was not possible because of the small number of studies.

While LLLT demonstrates promising therapeutic effects, the lack of standardized treatment protocols and the variability in study methodologies warrant further high-quality research. In fact, there is a lack of standardized protocols in the literature to use laser technology as an adjuvant to BRONJ. In the literature, other than surgery and pharmacological therapy, there are no other scientifically well-supported methods. Therefore, with further RCTs and clinical trials, LLLT could be used as the treatment of choice for BRONJ and avoid destructive interventions for the patient. Future large-scale randomized controlled trials (RCTs) with standardized laser parameters, longer follow-up durations, and well-defined outcome measures are necessary to validate the efficacy of LLLT and its role in the management of BRONJ. Additionally, further studies should explore the underlying mechanisms of LLLT in bone regeneration and its long-term impact on patient outcomes.

Overall, this review highlights the potential of LLLT as an adjunct in BRONJ treatment but underscores the necessity of more robust evidence to establish its clinical utility with greater confidence. A multidisciplinary approach integrating surgical, pharmacological, and laser-assisted strategies may provide a more comprehensive and effective treatment paradigm for BRONJ patients, ultimately improving their quality of life and clinical prognosis.

## Figures and Tables

**Table 1:** Search strategy.

***PubMed*** (“laser therapy”[MeSH Terms] OR (“laser”[All Fields] AND “therapy”[All Fields]) OR “laser therapy”[All Fields]) AND (“bisphosphonated”[All Fields] OR “bisphosphonic”[All Fields] OR “diphosphonates”[MeSH Terms] OR “diphosphonates”[All Fields] OR “bisphosphonate”[All Fields] OR “bisphosphonates”[All Fields]) AND (“medication-related”[All Fields] AND (“osteonecrosis”[MeSH Terms] OR “osteonecrosis”[All Fields] OR “osteonecroses”[All Fields]))
***Web of Science*** (Laser therapy) AND (Osteonecrosis) (ALL FIELDS)
***Scopus*** (Laser therapy) Palavras AND (Osteonecrosis)

**Table 2:** Summary of the findings of the included studies.

Title	Research Question	Main Findings
Bisphosphonates-related osteonecrosis of the jaws: a concise review of the literature and a report of a single-centre experience with 151 patients	What is the experience of the University of Parma with BPT patients?	- **Er:YAG laser + medical therapy** had the **highest improvement rate (96.55%)** and **complete healing (89.65%)**. - No significant link between healing and factors like BPT discontinuation or BRONJ location.
Bisphosphonate-related osteonecrosis: laser-assisted surgical treatment or conventional surgery?	Is laser-assisted surgery more effective than conventional surgery for BRONJ?	- **No significant difference** in treatment outcomes. - **Serum CTX levels** did not affect prognosis. - Stage II BRONJ had better healing rates than stage I.
Early Surgical Laser-Assisted Management of Bisphosphonate-Related Osteonecrosis of the Jaws (BRONJ): A Retrospective Analysis of 101 Treated Sites with Long-Term Follow-Up	What is the most effective treatment approach for ONJ in BPT patients?	- **Best results with Surgery + Laser + LLLT (89% improvement)**. - Surgical or combined therapy had higher healing rates than medical therapy alone. - **Early-stage (I & II) BRONJ responded better** than advanced stage III.
Surgical approach with Er:YAG laser on osteonecrosis of the jaws (ONJ) in patients under bisphosphonate therapy (BPT)	How effective is Er:YAG laser surgery for BRONJ patients?	- **87.5% complete mucosal healing**. - **100% of patients improved to a lower BRONJ stage**. - Less invasive, can be performed under **local anesthesia**, improving patient compliance.

Abbreviations: BPT (bisphosphonate therapy); BRONJ (bisphosphonate-related osteonecrosis of the jaw); ONJ (osteonecrosis of the Jaw); J/cm^2^ (Joule on centimeter), nm (nanometer), PBM (photobiomodulation).

**Table 3:** Additional specifics relate to the studies included in the literature.

Author(s)	Number of Patients	BRONJ Sites	Treatment Modalities	Key Findings
Vescovi et al. (2012) [55]	128	151	Medical therapy, surgical therapy, LLLT	Combined medical, surgical, and LLLT showed the highest success rate with respect to surgery only
Atalay et al. (2011) [57]	20	N/A	Laser-assisted surgery, conventional surgery	No significant difference between laser with respect to surgery only.
Vescovi et al. (2012) [56]	151	192	Medical, surgical, LLLT, Er:YAG laser	Er:YAG laser + LLLT provided superior clinical improvement with respect to antibiotic therapy
Vescovi et al. (2010) [54]	91	115	Medical, medical therapy-LLLT, surgery, surgical-LLLT	Er laser associated with LLLT is more efficient with respect to surgery only

Abbreviations: LLLT (low-level laser therapy).

**Table 4:** Grading of evidence.

Certainty Assessment							No. of Patients		Effect		Certainty	Importance
No. of Studies	Study Design	Risk of Bias	Inconsistency	Indirectness	Imprecision	Other Considerations	[Intervention]	[Comparison]	Relative (95% CI)	Absolute (95% CI)		
New Outcome												
4	non-randomized studies	serious ^a^	serious	not serious	serious	all plausible residual confounding would suggest spurious effect, while no effect was observed	30/57 (52.6%)	86/55 (156.4%)	not estimable		⨁⨁◯◯ Low ^a^	IMPORTANT
								0.0%				

CI: confidence interval. Explanations: ^a^ The studies included utilized several methods in the control group. ⨁ absence of bias; ◯ presence of bias.

## Supplementary Materials

The following supporting information can be downloaded at: https://www.mdpi.com/article/10.3390/jcm14134441/s1, PRISMA 2020 Checklist.
